# Influence of black garlic and *Hypericum scabrum* on growth performance, fecal microbiota, diarrhea, oxidative stress and immune status in pre-weaning calves

**DOI:** 10.1007/s11250-026-05189-1

**Published:** 2026-07-08

**Authors:** Veysel Fatih Ozdemir

**Affiliations:** Department of Animal Science, College of Agriculture, Atatürk University, Erzurum, 25240 Türkiye

**Keywords:** Black garlic, Hypericum scabrum, Pre-weaning calves, Growth performance, Fecal microbiota, Diarrhea, Oxidative stress, Immune response

## Abstract

This study investigated the effects of supplementing milk with fermented black garlic (G) and *Hypericum scabrum* decoction (H) on growth performance, immune status, diarrhea incidence, inflammatory responses, oxidative stress, and gut microbiota in 28 pre-weaning Holstein calves. Blood WBC counts were significantly higher in the control (C) group than in the treatment groups (*P* *= 0.007*). At 45 days of age, CAT activity was significantly higher in the C group as compared to experimental calves. Concentration of IL-6 was significantly lower in the treatment groups (*P* < *0.01*). Regarding gut health, fecal scores significantly improved in supplemented groups, particularly on day 45 (*P* < *0.05*), and fecal *E. coli* counts were reduced by 10.7% in the GH group (*P* *= 0.043*). Serum IgA was significantly higher in the GH group (*P* *= 0.044*), suggesting a more robust first-line immune defense. However, a selective antimicrobial effect was observed, as *bifidobactera* levels also decreased by 9.8% and 8.7% in G and in GH groups as compared to C (*P* *= 0.024*). No significant differences were observed between groups in live weight and body measurements throughout the experimental period, except for in heart girth on day 60 (*P* < *0.05*). Starter consumption did not differ between groups, furthermore, the C group had higher FCR and group H had higher ADG than the C group during the 0–60-day period, and no difference was observed in FCR and ADG during other periods In conclusion, while fermented black garlic and *H. scabrum* support gastrointestinal health and modulate inflammatory responses, their impact on overall growth performance remains limited under the studied conditions, and the reduction in beneficial *bifidobacterium* warrants careful long-term evaluation.

## Introduction

The calves are the future of cattle enterprises; thus, the healthy rearing of newborn calves is crucial for milk production and profitability. Newborn calves are particularly susceptible to health problems such as diarrhea, infection, and oxidative stress during the first few weeks as their immune systems are underdeveloped (Du et al. 2023, Gökçe et al. 2025). Diarrhea is one of the most common problems encountered in the neonatal period, leading to fluid and electrolyte loss, growth retardation, and even mortality (Dratwa-Chałupnik et al. 2012). Moreover, diseases during the preweaning period can negatively affect the future reproductive performance and first-lactation milk production of heifers (Abuelo et al. 2021). Furthermore, increased oxidative stress and an uncontrolled inflammatory response can impair calf growth performance and hinder effective immune function, thereby increasing disease susceptibility (Abuelo 2025). Traditionally, antibiotics have been widely used to manage these health problems (Allam et al. 2024). However, the overuse of antibiotics poses risks to both animal and human health, necessitating the development of safe, natural alternative strategies.

In this context, herbal products, particularly *Allium sativum* (garlic) and *Hypericum species*, rich in biologically active compounds, have been widely studied in recent years as natural alternatives to traditional antimicrobial and immunomodulatory treatments. Black garlic, a product obtained through controlled fermentation and heat treatment, contains higher amounts of S-allyl cysteine, polyphenols, and various organosulfide compounds than fresh garlic (Ahmed and Wang 2021). This fermentation process increases the bioavailability of garlic and significantly increases its antioxidant capacity (Najman et al. 2020). Several studies indicate that garlic (*Allium sativum*) can regulate the gut microbiota, increase gastrointestinal motility, and reduce diarrhea (Ghosh et al. 2011). They may strengthen the immune system in young calves by stimulating immune responses (Kekana et al. 2020). Similarly, *Hypericum scabrum* is a medicinal plant noted for its rich bioactive compounds (Öner et al. 2025). Recent studies on *H. scabrum* have revealed that the plant possesses significant anti-inflammatory, antioxidant, antifungal, and cytotoxic effects (Ergin et al. 2022). The combined use of black garlic and *H. scabrum* is hypothesized to exert a complementary and synergistic effect, in which organosulfur compounds from garlic primarily target the microbial load and the gut microbiota, while polyphenols from *Hypericum* modulate inflammatory and oxidative stress pathways.

We hypothesize a conceptual model in which the bioactive polyphenols and organosulfide compounds from black garlic and *H. scabrum* modulate the nuclear factor of kappa light chain enhancer (NF-κB) signaling pathway. By inhibiting NF-κB activation, these compounds are expected to downregulate the production of pro-inflammatory cytokines (Surai et al. 2019; Vecchi et al. 2025), thereby alleviating oxidative stress and strengthening gut barrier function by reducing mucosal permeability (Omonijo et al. 2018). An improved intestinal barrier reduces the incidence and severity of diarrhea, ultimately diverting energy from immune activation toward growth, thereby improving FCR in calves (Özkaya et al. 2023).

While the individual effects of both herbal products have been investigated in various studies, there is very limited information regarding their combined use. Furthermore, studies that assess gut health, inflammation, oxidative stress, and performance together are rare. Therefore, investigating the synergistic effects of black garlic and *H. scabrum* on these signaling pathways and physiological outcomes will provide important scientific contributions to understanding their potential as bioactive alternatives to antibiotics in calf rearing.

## Materials and methods

### Plant material and sample preparation

Garlic samples (*Allium sativum L*.) used in this study were obtained from Taşköprü (Kastamonu, Türkiye) and prepared following the procedure previously described by Deniz et al. (2024). Briefly, fresh Taşköprü garlic was harvested from local farmer fields, shade-dried, and stored at + 4 °C until use. Fermented and dried garlic samples (black garlic) were prepared under controlled conditions (60 °C for 30 days), as reported by Deniz et al. (2024).

The dose of black garlic (400 mg/kg BW) was selected based on previous animal studies reporting biologically active and safe ranges of garlic supplementation (100–500 mg/kg BW), where improvements in growth performance, immune response, and gut microbiota modulation were observed in ruminants (Ghosh et al. 2011; Jagota et al. 2024; Martin and Chaudhry 2024). Given that fermentation enhances the bioavailability and antioxidant capacity of garlic by increasing levels of S-allyl cysteine and polyphenols, this dose was considered both safe and pharmacologically effective (Ahmed and Wang 2021; Najman et al. 2020). In the combination group, the dose was reduced to 200 mg/kg BW to evaluate potential synergistic effects while preventing excessive exposure to phytochemicals.

*Hypericum scabrum* aerial parts were collected in 2025 from the Palandöken region (Erzurum, Türkiye). The plant material was shade-dried and ground into a homogeneous powder. Aqueous decoctions were prepared by weighing 112 g of the dried aerial parts and extracting with 1 L of water. The mixture was heated to boil and maintained at a rolling boil for 3 h. After cooling to room temperature, the decoction was filtered to remove plant residues and the filtrate was used for administration. All decoctions were freshly prepared daily and were not stored.

Based on this preparation, the resulting decoction corresponded to approximately 112 mg/mL crude plant material equivalent. Accordingly, the administered dose of 40 mL/day provided approximately 4.48 g/day of dried plant equivalent per animal (and 2.24 g/day in the combination group).

Since specific dosing studies for *H. scabrum* are limited, dose selection was guided by data from related *Hypericum* species, particularly *Hypericum perforatum*, which is extensively documented in herbal monographs such as the European Medicines Agency (2015), Commission (1998), and the European Scientific Cooperative on Phytotherapy (ESCOP 2013). These monographs indicate that daily human consumption of *Hypericum* corresponds approximately to 2–4 g of dried plant material (or equivalent extracts), typically administered as infusions or decoctions. When scaled to body weight and considering interspecies differences, the dose used in this study falls within a biologically relevant and pharmacologically active range. Furthermore, previous animal studies using *Hypericum* extracts have demonstrated antioxidant, anti-inflammatory, and immunomodulatory effects at comparable plant equivalent doses (Bombik et al. 2012; Ilhan et al. 2024). Accordingly, a daily dose of 40 mL per animal was selected to ensure sufficient exposure to bioactive compounds. In the combination group, the dose was reduced to 20 mL/day to maintain balance in total phytochemical intake.

Importantly, the selected dose was sufficient to ensure exposure to bioactive compounds such as flavonoids and phenolic acids, which contribute to the observed biological effects, while remaining within a safe range supported by both traditional use and experimental studies.

### LC–Orbitrap HRMS analysis of phenolic compounds

The phenolic composition of the solid extract samples was analyzed by liquid chromatography-high resolution mass spectrometry (LC–HRMS) at the Central Research and Application Laboratory of Bingöl University (Bingöl, Türkiye). Analyses were carried out using a Thermo Scientific Exactive Plus Orbitrap™ mass spectrometer equipped with a heated electrospray ionization (HESI) source and coupled to a Dionex UltiMate™ 3000 RS UHPLC system. Chromatographic separation was performed on a COSMOSIL 3C18-EB column (100 × 2.0 mm) using a gradient system of 0.5% (v/v) acetic acid in ultrapure water and LC–MS grade methanol at a flow rate of 0.30 mL min⁻¹, with a 20 µL injection volume and a total run time of 20 min. The Orbitrap MS was operated in both positive and negative ion modes over an m/z range of 60–800, and data acquisition and processing were performed using TraceFinder™ and Xcalibur™ software. Quantification was achieved using external calibration curves constructed from phenolic standards (91 compounds) in the range of 10–1000 ppb, injected in triplicate. Before analysis, samples were filtered through 0.22 μm PTFE syringe filters and transferred to LC vials for injection.

### Animals, housing and experimental design

In this study, 28 female Holstein Friesian calves born at the Cattle Breeding Unit of the Atatürk University Food and Livestock Application and Research Center were used (7 calves in each group). Ethical approval for the study was obtained from the Atatürk University Local Animal Experiments Ethics Committee under decision No. 158, dated 24 June 2024 (Session 2024/06).

After birth, the calves were allowed to spend 3 days in the same compartment but different pens with their dams for colostrum feeding. After three days of colostrum feeding, the calves were moved to a calf barn with individual pens (1.2 m x 2.0 m). The pens were bedded with clean wheat straw, which was refreshed daily. The barn was maintained with natural ventilation within the thermoneutral temperature range (15–22 °C). Since the study was conducted at a university research and application farm, all animals were subject to a strict, continuous health-monitoring protocol. In accordance with the center’s standard operating procedures, calves are clinically inspected at least twice weekly by two staff veterinarians for rectal temperature, respiratory rate, nasal discharge and other signs. Calves were randomly allocated into four groups coded as G (black garlic, *n* = 7), H (*hypericum*, *n* = 7), GH (black garlic + *hypericum* combination, *n* = 7), and C (control, *n* = 7), based on their birth order and initial body weight to ensure group homogeneity. The supplements were added directly to the morning milk for the treatment groups and administered once daily in the following doses. Garlic supplement dosages were determined based on individual body weight (BW) and were recalculated and adjusted every 2 weeks after weighing.

- C Group: Only milk.
- G Group: Milk supplemented with 400 mg/kg BW Black garlic.
- H Group: Milk supplemented with 40 mL/day per animal *H. scabrum* decoction.
- GH Group: Milk supplemented with a mixture of 200 mg/kg BW Black garlic + 20 mL/day per animal *H. scabrum* decoction.

The garlic preparation given to calves in the garlic group was added to the milk, to create a homogeneous mixture, then blended with a blender. In the *Hypericum* group, a water-based *H. scabrum* extract was prepared daily and added to the milk before the millk was fed to the calves.

Supplements were freshly prepared each day, with strict hygiene maintained during their administration. All containers, blenders, and measuring equipment were thoroughly cleaned before and after use. Calves received milk at 10% of their birth weight, divided into two daily feedings, until weaning at 60 days of age. Starter feed was provided once daily at 8:00 am, clean water and starter feed were continuously available throughout the study. Daily starter consumption was monitored daily by recording the amount offered and weighing the remaining feed from the previous day.

### Live weight and body measurements

To determine the calves’ growth performance and body development, live weight and various body measurements were taken starting at 3 days of age and repeated on days 15, 30, 45, and 60 days of age. An electronic scale was used to measure live weight. Heart girth, withers height and body length were determined using a tape measure and a measuring stick. To minimize errors, all measurements were taken by the same person.

### Fecal consistency scoring

The fecal consistency of calves was assessed at 15-day intervals using the scoring system developed by Larson (1977). According to this method, fecal consistency was classified as follows: score 1 = Normal (soft solid, not runny); 2 = Soft (mostly solid, partly soft); 3 = Loose (semi-liquid, predominantly liquid); 4 = Watery (completely liquid).

### Microbiology

Fecal samples were aseptically collected from the calves’ rectum at 2 and 8 weeks of age and transported to the laboratory in sterile containers under cold chain conditions. Analyses were performed immediately following arrival. Total bacterial counts were determined using PCA agar, *lactic acid bacteria* with MRS agar, *E. coli* with TBX agar, total *coliforms* with VRBD agar, and *bifidobacteria* with BFD agar. Appropriate serial dilutions were prepared for each sample, plated on the respective media, and incubated at 37 °C for 24–48 h in accordance with standard protocols. Colony counts were conducted using established microbiological techniques, and all values were logarithmically transformed (log₁₀).

### Blood sampling

Blood samples were obtained from the calves’ jugular vein at 3, 15, 30, 45, and 60 days of age. For serum collection, samples were transferred into 8.5 mL gel serum tubes without anticoagulant and centrifuged at 3000–4000 rpm for 10 min to separate serum from blood cells. The serum was aliquoted and stored at − 80 °C until further analysis. For hematological analysis, blood samples were collected in K2EDTA tubes.

### ELISA analyses

Immunoglobulin levels (IgA, IgG, IgM), antioxidant enzyme activities (SOD, CAT, GSH-Px), and pro-inflammatory cytokine levels (IL-6, TNF-α, TNF-γ) were evaluated in serum samples from calves using ELISA, according to the manufacturer’s instructions (Sunred, China).

### Hematological analysis

The amounts of WBC (Leukocyte), LYM (Lymphocyte), MON (Monocyte), NEU (Neutrophil), and EOS (Eosinophil) were determined by the Abacus Junior Vet5 hemogram device in the fresh blood samples.

### Statistical analysis

The sample size for the study was constrained by practical considerations related to animal availability and experimental conditions. Following the experiment, a post-hoc power analysis was conducted using G*Power software (v3.1.9.7). Based on a repeated measures design and an assumed medium effect size (f = 0.25), a significance level of α = 0.05, a total sample size of 28, and five repeated measurements, the achieved statistical power (1 − β) was approximately 75.04%.

To determine the normal distribution of the data kurtosis and skewness values were calculated. Values between − 1.5 and + 1.5 for skewness and kurtosis were considered compatible with a normal distribution. Data were analyzed using three approaches, depending on the nature and distribution of the parameters. Longitudinal data were analyzed using a Linear Mixed Model (LMM) to account for repeated measures (Harrison et al. 2018). In this model, treatment, time, and their interaction were included as fixed effects, while each individual calf was modeled as a random effect with an autoregressive (AR1) covariance structure. Pairwise comparisons for interaction effects were performed using the Compare command with Bonferroni adjustment.

In contrast, performance parameters (starter intake, ADG, and FCR) calculated for specific periods were analyzed using One-Way ANOVA. When significant differences were detected, Duncan’s multiple range test was used for post-hoc comparisons. Fecal consistency scores, which were non-normally distributed and categorical, were analyzed using the non-parametric Kruskal–Wallis test. When significance was achieved in the Kruskal–Wallis analysis, pairwise comparisons between groups were performed using the Mann–Whitney U test. CFU/g values obtained from microorganism counts were normalized by log₁₀ transformation to ensure normal distribution and variance homogeneity assumptions.

## Results

Table 1 presents the phenolic composition of the black garlic sample (1 mg/mL in methanol) as determined by LC–HRMS analysis. Among the identified compounds, chlorogenic acid was the predominant phenolic constituent (13036.01 ng/mL), followed by protocatechuic acid (2269.72 ng/mL), catechin (1867.66 ng/mL), and quercetin (1864.34 ng/mL). Moderate amounts of 3-(4-hydroxyphenyl) propionic acid, gallic acid, quinic acid, and 4-hydroxybenzoic acid were detected, with concentrations ranging between approximately 300 and 650 ng/mL. Several flavonoids, including myricetin, kaempferol, luteolin, orientin, and isoorientin, were present at relatively low levels, whereas chrysin, apigenin, acacetin, and caffeic acid phenethyl ester (CAPE) were detected only in trace amounts (< 10 ng/mL). Overall, the phenolic profile of the garlic sample was dominated by phenolic acids, with chlorogenic acid representing the major compound.

**Table 1:** Phenolic compound content of fermented garlic used in the study

Phenolic Compounds	Amount (ng/mL)		Phenolic Compounds	Amount (ng/mL)
4-Hydroxybenzoic acid	341.62		3-(4-Hydroxyphenyl) propionic acid	648.55
Salicylic acid	171.30		Catechin (Cianidanol)-p	1867.66
Syringic acid	13.526		Epigallocatechin	43.70
Gallic acid (3,4,5-trihydroxybenzoic acid)	619.28		Chrysin (5,7-Dihydroxy-2-phenyl-4 H-chromen-4-one)	1.65
Protocatechuic acid (3,4-Dihydroxybenzoic acid)	2269.72		Apigenin (5,7-Dihydroxy-2-(4-hydroxyphenyl)-4 H-chromen-4-one)	3.49
3,4-dihydroxybenzaldehyde (Protocatechuic aldehyde)	1.528		Acacetin (5,7-Dihydroxy-2-(4-methoxyphenyl)-4 H-chromen-4-one)	1.77
2,4-dihydroxybenzoic acid (beta-Resorcylic acid)	97.56		Vicenin 2	133.47
Vanillic acid	68.17		Luteolin	32.24
Gentisic acid	174.73		Diosmetin (Luteolin 4′-methyl ether)	2.73
trans Cinnamic acid	56.30		Orientin	64.83
Caffeic acid	74.15		Isoorientin	66.54
Caffeic acid phenhyl ester (CAPE)	0.83		Galangin (3,5,7-Trihydroxy-2-phenyl-4 H-chromen-4-one)	5.89
Ferulic acid	52.70		Quercetin	1864.34
Sinapic acid	36.28		Kaempferol	37.46
Chlorogenic acid	13036.02		Kaempferide	9.01
Quinic acid	341.43		Myricetin	247.76

Table 2 summarizes the phenolic compound content of *H. scabrum* used in the study as determined by LC–HRMS analysis. Among the detected compounds, quercetin was the most abundant phenolic constituent (45.88 ng/mL), followed by vitamin C (22.28 ng/mL), isoquercitrin (21.16 ng/mL), ellagic acid (17.61 ng/mL), and myricetin (17.58 ng/mL). Lower concentrations of caffeic acid, kaempferide, galangin, and benzoic acid were observed, whereas chrysin and afzelin were detected only in trace amounts (< 1 ng/mL). Overall, the phenolic profile of the *H. scabrum* sample was characterized by a predominance of flavonols and their glycosylated derivatives.

**Table 2:** Phenolic compound content of *H. scabrum* used in the study

Phenolic Compounds	Amount (ng/mL)
Benzoic acid	1.12
Caffeic acid	2.91
Chrysin (5,7-Dihydroxy-2-phenyl-4 H-chromen-4-one)	0.31
Galangin (3,5,7-Trihydroxy-2-phenyl-4 H-chromen-4-one)	1.18
Quercetin	45.88
İsoquercitrin (Quercetin 3-glucoside)	21.16
C vit	22.28
Afzelin (Kaempferol 3-rhamnoside)	0.63
Kaempferide	2.39
Myricetin	17.58
Ellagic acid	17.61

**Fig. 1:**
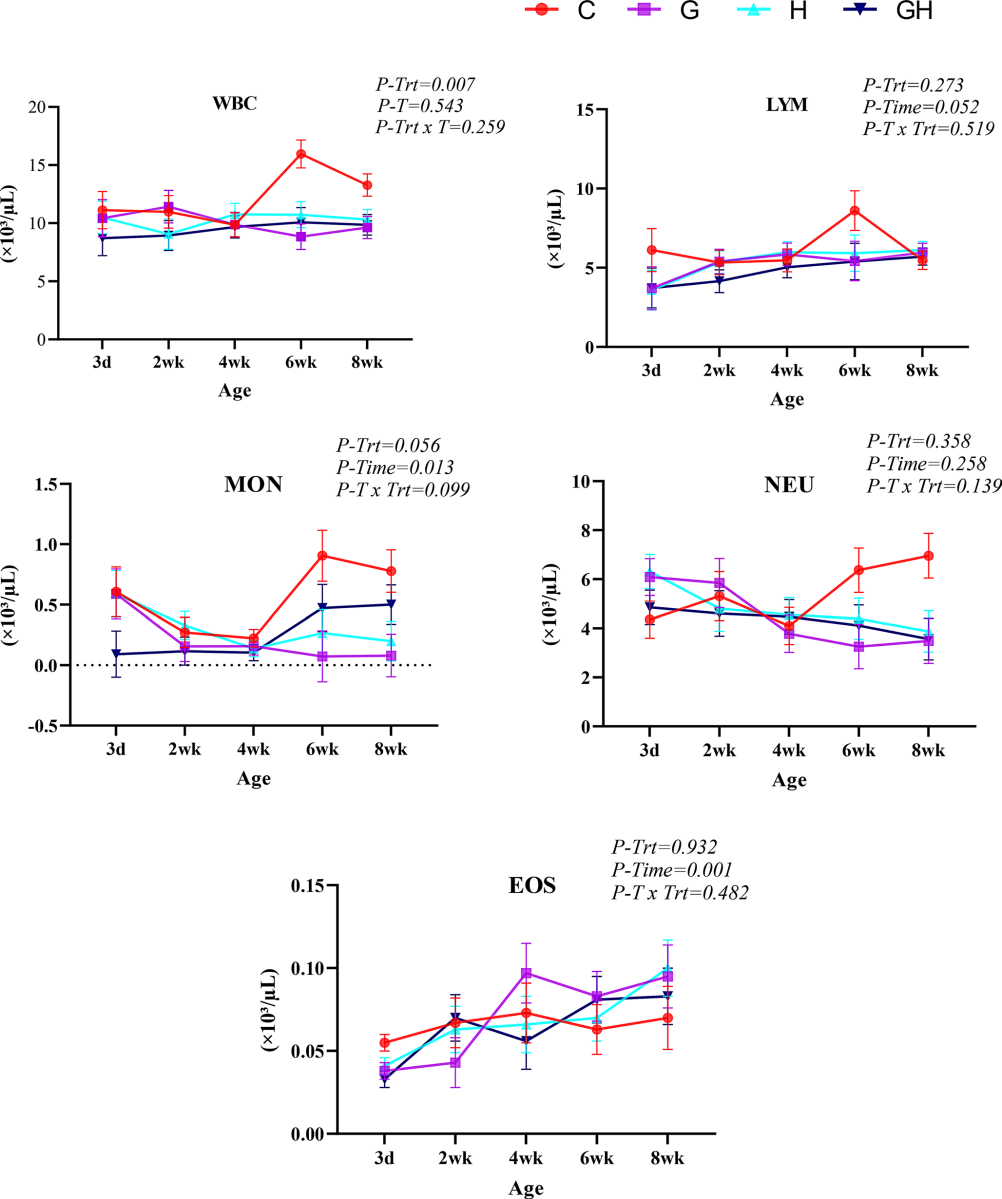
Some hematological parameters of pre-weaning calves. Blood samples were collected at 3, 15, 30, 45 and 60 days of age. Data are presented as means ± SEM. P-values for treatment (P-Trt), time (P-Time), and their interaction (P-Trt × T) are provided for each parameter. Abbreviations: d, day; EOS, eosinophil; LYM, lymphocyte; MON, monocytes; NEU, neutrophil; WBC, white blood cell; wk, week; µL, microliter

Figure 1 shows the results of hematological parameters of calves. There was a significant effect of treatment on WBC counts (*P* *= 0.007*). Specifically, the C group showed a noticeable increase at 6 wk compared to the treatment groups. However, the time effect (*P* *= 0.543*) and treatment × time interaction (*P* *= 0.259*) were not significant. A significant time effect was observed (*P* *= 0.013*) in MON counts, while the treatment effect was near the significance threshold (*P* *= 0.056*). Similarly, EOS counts were significantly affected by time (*P* *= 0.001*), showing a general increase as the calves aged, but no treatment differences were detected (*P* *= 0.932*). No significant treatment, time, or interaction effects were observed for LYM and NEU counts (*P* > *0.05).*

**Fig. 2:**
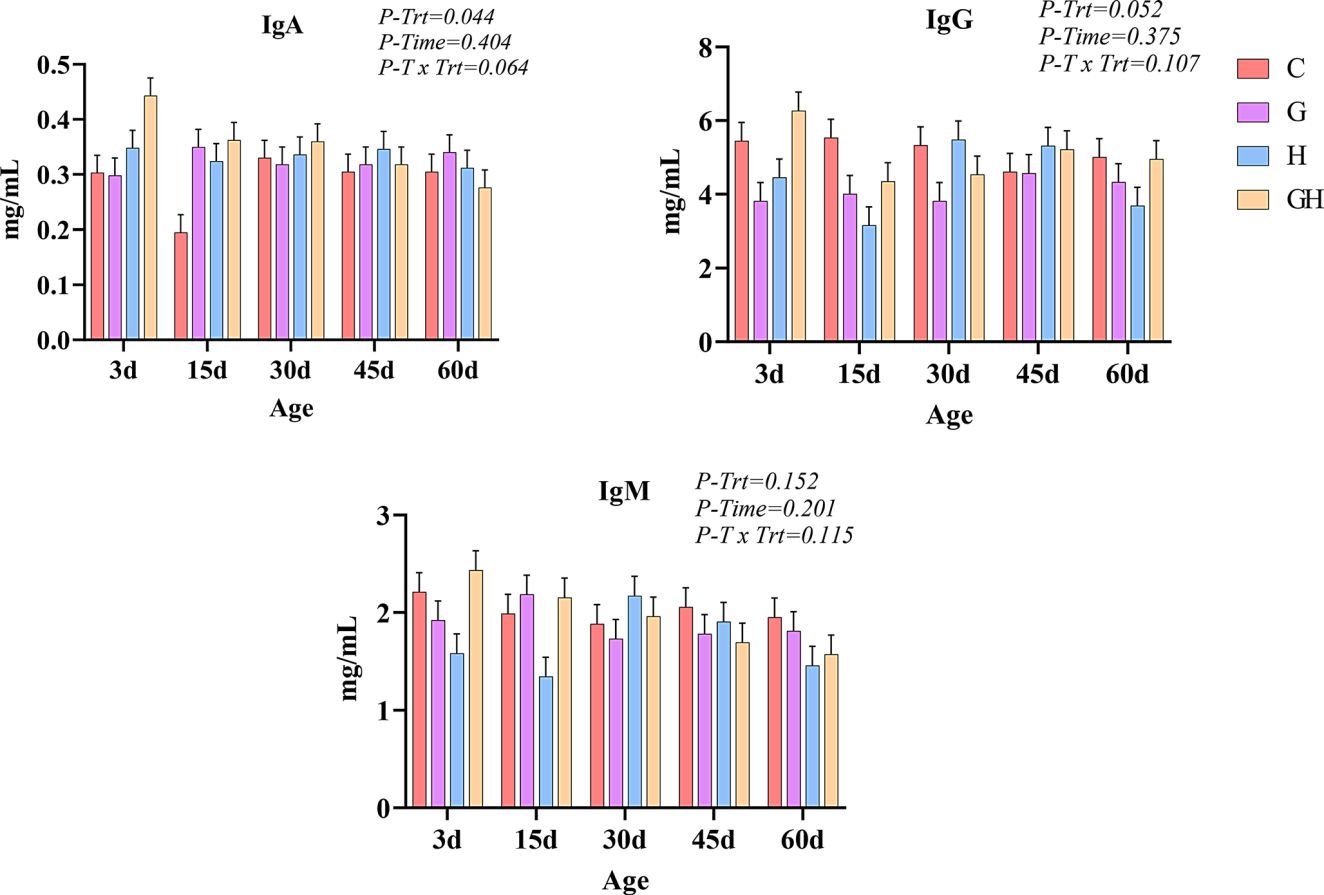
Serum Immunoglobulin levels of pre-weaning calves. Blood samples were collected at 3, 15, 30, 45 and 60 days of age. Data are presented as means ± SEM. P-values for treatment (P-Trt), time (P-Time), and their interaction (P-Trt × T) are provided for each parameter. Abbreviations: d, day; Ig, Immunoglobulin; mg, milligram; mL, milliliter

Changes observed in serum immunoglobulin concentrations (IgA, IgG, and IgM) during the study are presented in Fig. 2. The effect of treatment on serum IgA levels was found to be statistically significant (*P* *= 0.044*), and the treatment × time interaction was at the limit of significance (*P* *= 0.064*). Post-hoc analyses revealed a statistically significant difference between the C and GH groups. In contrast, no statistically significant effect of treatment, time, or their interaction was found on serum IgG and IgM levels (*P* > *0.05*).

**Fig. 3:**
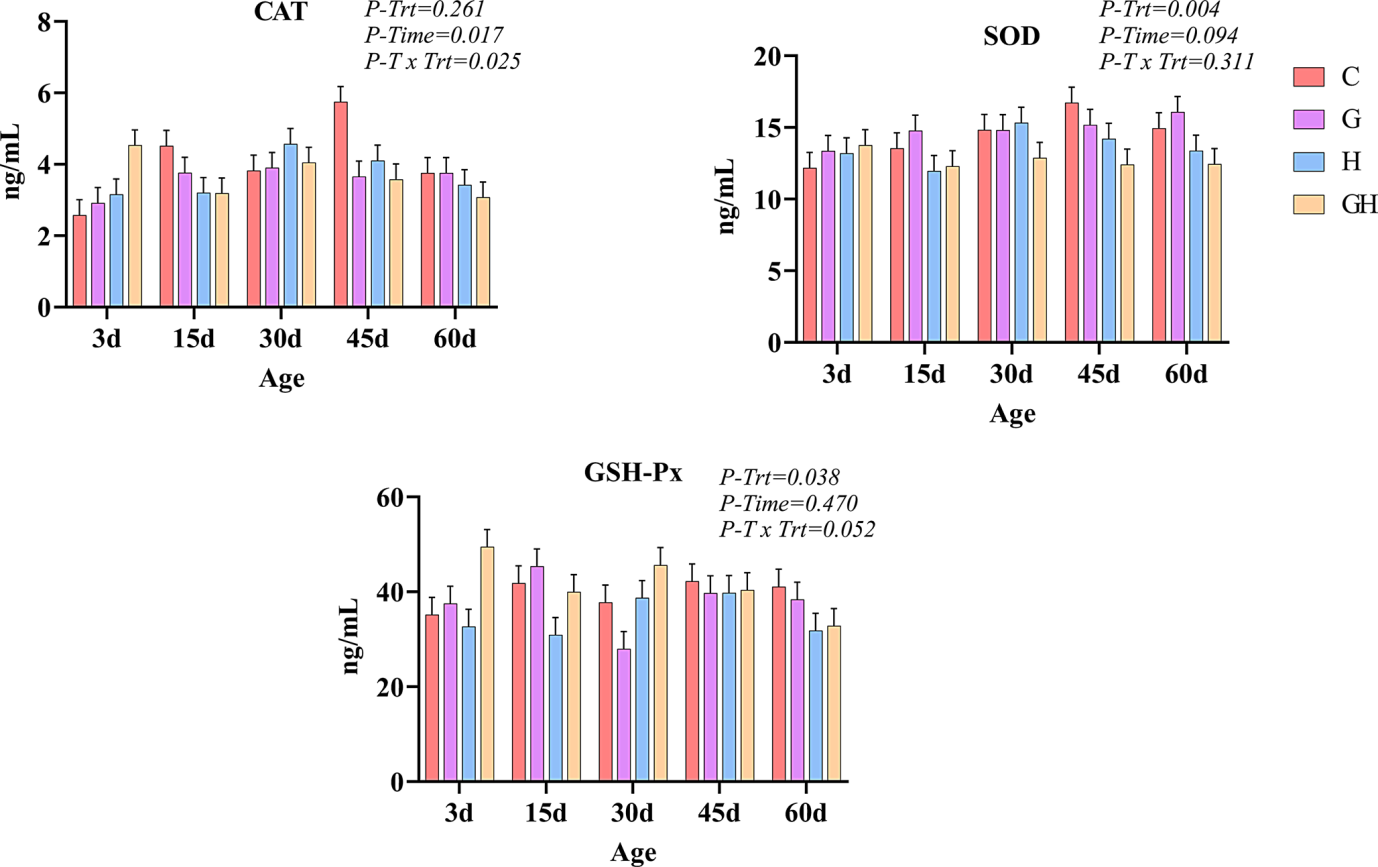
Antioxidant enzyme activities in pre-weaning calves. Blood samples were collected at 3, 15, 30, 45 and 60 days of age. Data are presented as means ± SEM. P-values for treatment (P-Trt), time (P-Time), and their interaction (P-Trt × T) are provided for each parameter. Abbreviations: d, day; Ig, Immunoglobulin, mg, milligram; mL, milliliter, CAT, Catalase; SOD, Super Oxide Dismutase, GSH-Px, Glutathione peroxidase

The results of serum antioxidant enzyme activities (CAT, SOD, and GSH-Px) during the study are presented in Fig. 3. The effect of time on CAT activities was statistically significant (*P* *= 0.017*), and a significant treatment × time interaction was also observed (*P* *= 0.025).* The CAT level of the C group on day 45 was significantly higher than that of the other groups. The effect of treatment on SOD activities was highly significant (*P* *= 0.004*), and post-hoc analyses revealed that SOD levels in the GH group were statistically lower than those of in C and G. In GSH-Px activities, the main effect of treatment was found to be significant (*P* *= 0.038*), and the treatment × time interaction was also at the limit of significance (*P* *= 0.052*); in intergroup comparisons, a statistically significant difference was found between group H and group GH, and it was noted that the GH group had higher GSH-Px activity than the H group, especially on day 3.

**Fig. 4:**
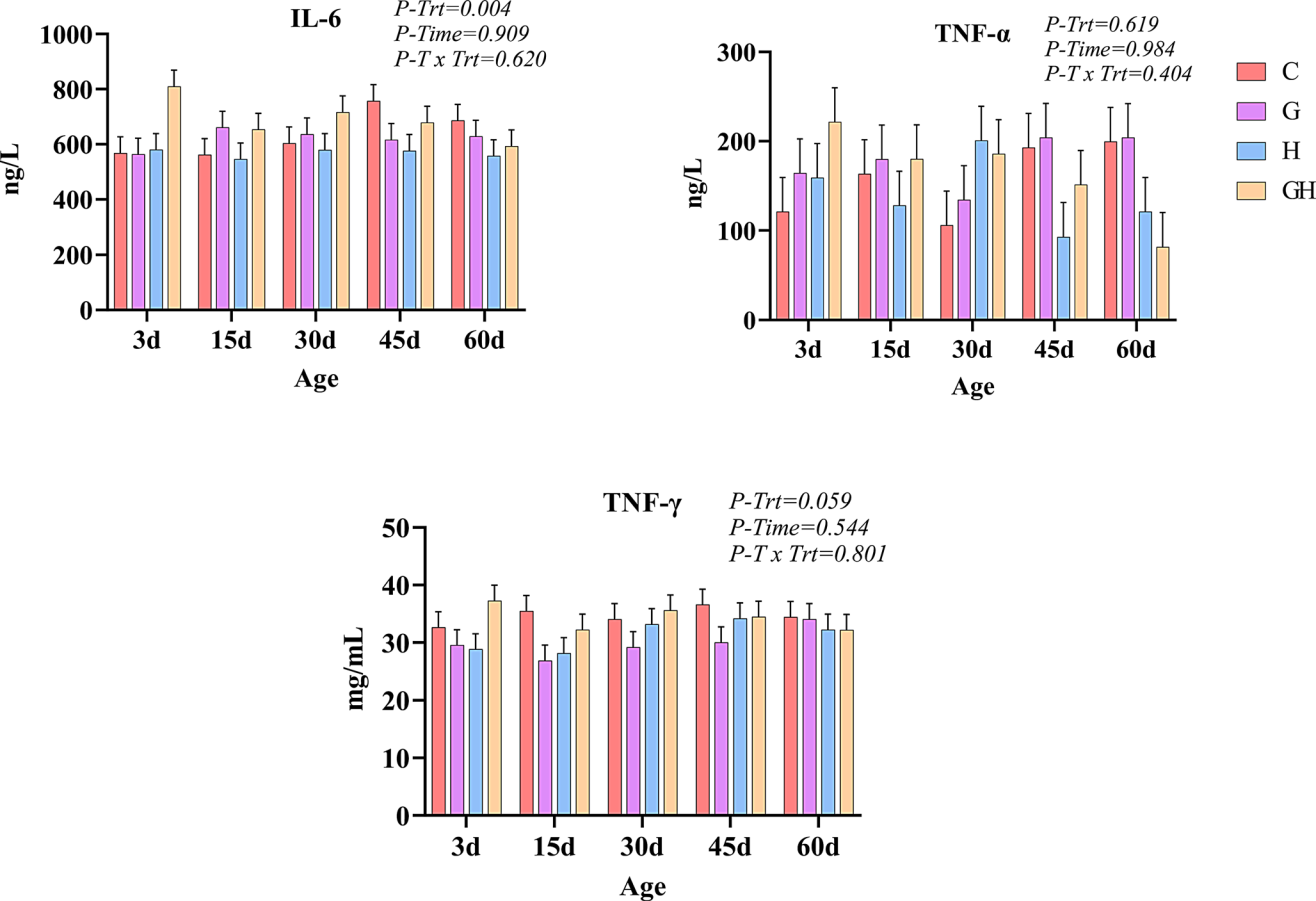
Serum pro-inflammatory cytokine levels in pre-weaning calves. Blood samples were collected at 3, 15, 30, 45 and 60 days of age. Data are presented as means ± SEM. P-values for treatment (P-Trt), time (P-Time), and their interaction (P-Trt × T) are provided for each parameter. Abbreviations: d, day; Ig, Immunoglobulin, mg, milligram; ng, nanogram; mL, milliliter, IL-6, Interleukin-6; TNF, Tumor Necrosis Factor

The results of serum cytokine profiles (IL-6, TNF-*α*, and TNF-*γ*) are presented in Fig. 4. A statistically significant effect of the treatment groups was detected on serum IL-6 levels (*P* *= 0.004*). Post-hoc comparisons revealed a distinct difference particularly between the H and GH groups. It was observed that calves in the GH group had higher IL-6 concentrations than those in the H group, particularly at the start of the study (day 3). In contrast, the H group exhibited more stable levels throughout the experimental period. Regarding TNF-α levels, no significant differences were found between groups, time points, or their interaction (*P* > *0.05*). For TNF-*γ* levels, a trend toward differences among groups was observed (*P* *= 0.059*); however, this did not reach statistical significance.

**Fig. 5:**
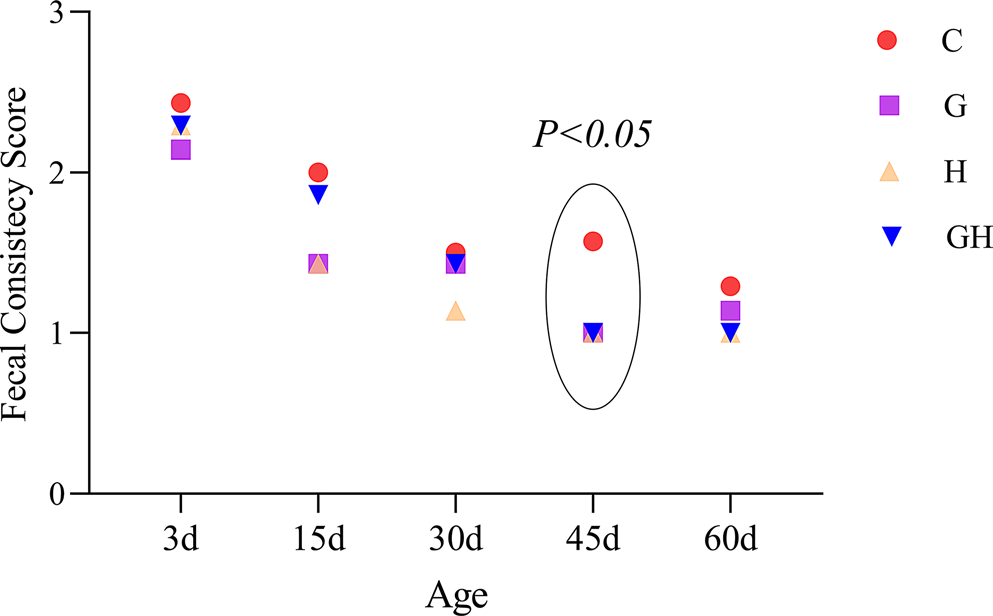
Fecal consistency scores of pre-weaning calves under four dietary treatments. The evaluations were conducted at 3, 15, 30, 45 and 60 days of age

As shown in Fig. 5, fecal scores of calves were monitored on days 3, 15, 30, 45, and 60 of age. Fecal consistency scores were generally higher in the C group throughout the monitoring period. Specifically, at 45 days of age, mean fecal scores of calves in the C group were statistically significantly higher than those in the other treatment groups (*P* < *0.05*).

**Fig. 6:**
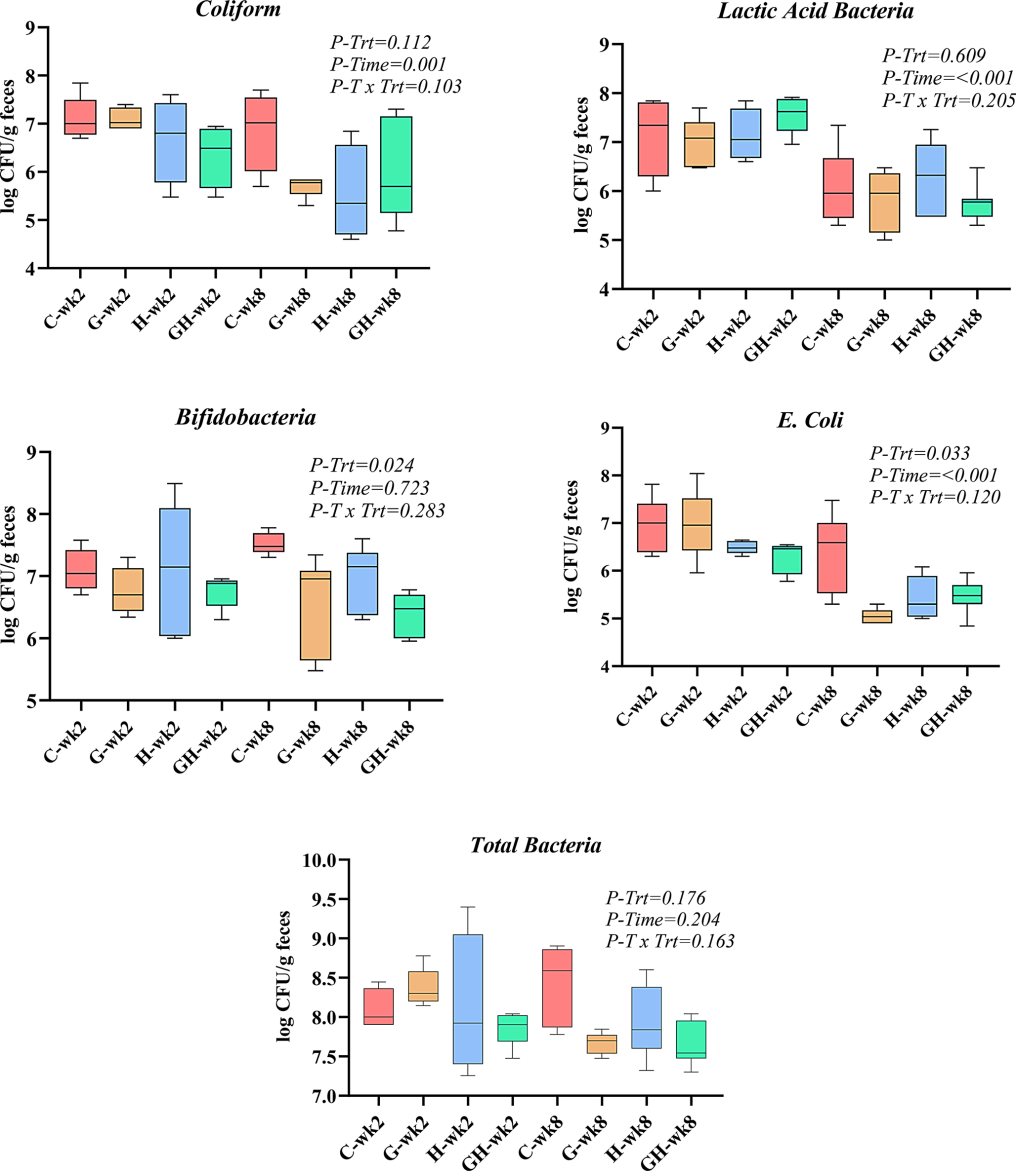
Fecal microbial counts in calves at 2 and 8 weeks of age under four dietary treatments. P-values for treatment (P-Trt), time (P-Time), and their interaction (P-Trt × T) are provided for each parameter. Abbreviations: wk, week; Trt, treatment; T, Time; CFU, Colony Forming Unit

Figure 6 shows microbial counts in fecal samples from calves at 2 and 8 weeks of age. Microbiological analyses revealed no statistically significant differences in *coliform*, *lactic acid bacteria and* total *bacterial* counts among groups. In contrast, significant differences were found in *E. coli* (*P* *= 0.033*) and *bifidobacteria* (*P* *= 0.024*) levels. Post-hoc pairwise comparisons revealed that the GH group had significantly lower *E. coli* and *bifidobacterial* counts than the C group. Additionally, a statistical trend toward lower *bifidobacterial* counts was observed in G group as compared to C group (*P* *= 0.099*).

The changes in body weight and measurements throughout the 8-week study period are presented in Table 3. As expected, a highly significant effect of time was observed for all growth parameters (*P* < *0.001*), reflecting the steady physiological development of the calves. A significant treatment × time interaction was detected in live weight (*P* *= 0.001*) Supplemented groups (G, H, GH) reached higher final weights (75.00–77.00 kg) compared to the C (69.50 kg) at 8 weeks. Similarly, Heart Girth exhibited a significant interaction (*P* *= 0.022*), where the G (96.67 cm) and H (96.57 cm) groups showed significantly higher values than the C (92.00 cm) group at the final measurement (*P* < *0.05*). In contrast, no significant treatment or interaction effects were found for Withers Height and Body Length (*P* > *0.05*), although numerical improvements were observed in the supplemented groups.

**Table 3:** Body measurements and live weight of calves from birth to weaning

Parameter	Age (day)	Groups				Overall	SEM	*P*		
		C	G	H	GH			*Trt*	*Time*	*Time x Trt*
Live Weight	3	39.23	40.50	40.00	38.14	39.46^A^	2.24		*< 0.001*	*0.001*
	15	42.50	45.17	44.14	42.71	43.63^B^				
	30	50.33	51.33	50.71	50.14	50.63^C^				
	45	58.17	61.5	63.14	60.57	60.84^D^				
	60	69.50	76.08	77.00	75.00	74.39^E^				
	Overall	51.95	54.92	55.00	53.31			*0.749*		
Withers Height	3	73.00	74.67	73.43	74.43	73.88^A^	1.07		*< 0.001*	*0.908*
	15	74.83	77.17	76.14	76.14	76.07^B^				
	30	77.83	79.83	78.71	79.14	78.88^C^				
	45	80.67	82.00	80.86	82.14	81.42^D^				
	60	83.83	85.83	84.71	85.14	84.88^E^				
	Overall	78.03	79.90	78.77	79.40			*0.611*		
Body Length	3	68.50	69.83	67.86	67.14	68.33^A^	1.19		*< 0.001*	*0.580*
	15	71.00	72.33	70.29	69.85	70.87^B^				
	30	73.67	75.17	73.71	74.28	74.21^C^				
	45	76.33	83.33	77.57	77.00	77.31^D^				
	60	79.33	81.67	80.71	80.71	80.71^E^				
	Overall	73.77	75.47	74.11	73.80			*0.704*		
Heart Girth	3	77.16	78.67	76.71	77.00	77.39^A^	1.13		*< 0.001*	*0.022*
	15	79.00	81.66	79.17	80.00	79.95^B^				
	30	82.67	85.33	84.14	83.86	84.00^C^				
	45	87.17	91.33	89.14	88.14	88.95^D^				
	60	92.00^b^	96.67^a^	96.57^a^	94.57^ab^	94.95^E^				
	Overall	83.60	86.73	85.14	84.71			*0.279*		

*a, b: Means within a row with different small superscripts differ significantly between treatment groups (*P* < *0.05*). A, B: Means within a column with different capital superscripts differ significantly between time points within the same group (*P* < *0.05*)

Abbreviations: d, day; kg, kilogram; wk, week; T, Time; Trt, Treatment; SEM, Standard Error Mean

Table 4 presents the starter consumption, FCR and average ADG of calves between 0 and 60 days of age. Starter consumption did not differ significantly between groups across periods (*P* > *0.05*). The C group showed higher FCR values compared to the G and H groups during the 30–45-day period (*P* < *0.01*). Significant differences were also found between the groups in terms of ADG during the 30–45-day period (*P* < *0.01*). Moreover, FCR of calves in the C group was significantly higher than other groups for the 0–60 days of age period. The ADG value of the H group was higher than that of the C group. No significant differences were observed between the groups for both FCR and ADG during the other periods.

**Table 4:** Performance parameters of calves from birth to weaning under four dietary treatments

		0–15 days	15–30 days	30–45 days	45–60 days	0–60 days
		Mean ± S.E.	Mean ± S.E.	Mean ± S.E.	Mean ± S.E.	Mean ± S.E.
Starter Intake (kg)	C	0.22 ± 0.04	3.59 ± 0.48	8.04 ± 1.28	13.26 ± 1.87	25.11 ± 3.52
	G	0.19 ± 0.10	3.88 ± 0.88	9.53 ± 1.50	13.05 ± 1.16	26.65 ± 3.16
	H	0.22 ± 0.07	2.94 ± 0.38	8.29 ± 0.81	13.10 ± 0.99	24.55 ± 1.63
	GH	0.26 ± 0.05	2.69 ± 0.33	6.48 ± 0.80	12.31 ± 0.59	21.75 ± 1.26
	*P*	*0.932*	*0.380*	*0.297*	*0.942*	*0.555*
Feed Conversion Ratio	C	2.42 ± 0.22	1.49 ± 0.18	2.01 ± 0.22^a^	1.85 ± 0.21	1.80 ± 0.10^a^
	G	1.73 ± 0.15	1.88 ± 0.08	1.75 ± 0.22^ab^	1.42 ± 0.08	1.60 ± 0.07^ab^
	H	2.29 ± 1.43	1.66 ± 0.16	1.28 ± 0.07^b^	1.55 ± 0.11	1.51 ± 0.10^b^
	GH	2.04 ± 0.22	1.39 ± 0.12	1.31 ± 0.07^b^	1.35 ± 0.06	1.38 ± 0.06^b^
	*P*	*0.227*	*0.109*	***0.007***	*0.053*	***0.020***
Average Daily Gain (kg)	C	0.13 ± 0.02	0.52 ± 0.06	0.52 ± 0.03^c^	0.76 ± 0.04	0.50 ± 0.02
	G	0.31 ± 0.03	0.41 ± 0.04	0.68 ± 0.06^b^	0.97 ± 0.05	0.59 ± 0.03
	H	0.28 ± 0.05	0.44 ± 0.03	0.83 ± 0.05^a^	0.92 ± 0.09	0.62 ± 0.04
	GH	0.30 ± 0.02	0.50 ± 0.05	0.70 ± 0.04^ab^	0.96 ± 0.03	0.61 ± 0.03
	*P*	*0.244*	*0.323*	***0.002***	*0.073*	*0.062*

*a, b: Means within a row with different small superscripts differ significantly between treatment groups (*P* < 0.05). Abbreviations: kg, kilogram; SE, Standard Error

## Discussion

Supporting immunity, reducing oxidative stress, and maintaining a balanced gut microbiota are particularly important during the pre-weaning period, when calves are highly susceptible to diarrhea and other health challenges (Du et al. 2023; Li et al. 2025). Previous in vitro and in vivo studies have demonstrated that Protocatechuic acid which was found to be abundant in the fermented garlic used in this study, enhances antioxidant capacity, modulates immune and gut functions, suppresses pathogenic microorganisms, and thereby supports overall animal health and growth performance (Mahfuz et al. 2022). Hematological findings from this study indicate that WBC values were significantly lower across all treatment groups compared with the C group (*P* < *0.01*). This increasemay indicate an inflammatory response or subclinical stress in calves in the C group calves. Although previous studies have reported no significant effects of garlic on hematological parameters in young ruminants (Ikyume et al. 2017; Jagota et al. 2024), the black garlic used in our study may be effective in reducing inflammatory responses and stress indicators in the C group with its distinct bioactive composition. Furthermore, H and GH supplementation have resulted in decreased WBC and MON counts. Comparably, Bombik et al. (2012) reported that an herbal mixture containing *H. perforatum* caused significant decreases in WBC counts of Polish Holstein Friesian calves.

Plant antioxidants have been reported to support humoral immunity by increasing serum immunoglobulin levels (Kewan et al. 2021; Redoy et al. 2020). Phytogenic compounds such as garlic and fermented garlic, have beenreported to increase IgG and IgM levels (Ao et al. 2011; Wang et al. 2011; Fathi et al. 2025). In this study, no significant differences were found between the groups in terms of serum immunoglobulin levels (IgG and IgM). Conflicting results have been reported in calves and lambs by Kekana et al. (2020) and Kewan et al. (2021) using raw garlic. Few studies have investigated the use of *Hypericum* species on the Ig levels. In parallel with findings Aghili et al. (2014) reported no significant effect on IgG levels in rats with *H. perforatum* administration. However, serum IgA levels were significantly higher in the GH group, suggesting a potential enhancement of mucosal immune defense, which may be associated with the reduced fecal *E. coli* counts observed in this study.

Dietary supplementation with polyphenol-rich plant extracts has been shown to reduce oxidative stress, enhance antioxidant enzyme activities, and modulate inflammatory pathways in vivo. While effects on growth performance are variable, polyphenols consistently improve animal health, gut integrity, immune responses, and product quality by enhancing oxidative stability (Lipiński et al. 2017). In the present study, CAT and SOD activities were higher in the C group on day 45 compared to the experimental groups. Although elevated CAT and SOD activities are sometimes interpreted as indicators of increased oxidative challenge, such enzyme responses can also reflect adaptive or compensatory mechanisms of the antioxidant defense system (Surai et al. 2019). Therefore, the lower CAT and SOD activities observed in the supplemented groups should be interpreted cautiously, as they may reflect altered antioxidant enzyme regulation rather than a definitive reduction in oxidative stress. Since antioxidant enzyme activities alone are insufficient to comprehensively characterize oxidative status, additional oxidative biomarkers are needed to better clarify the effects of these supplements on redox balance. Previous studies in ruminants have reported that garlic and different *Hypericum* species can modulate antioxidant enzyme activities under oxidative challenge conditions (Yang et al. 2021; Zhou and Shen 2025; Hariharapura et al. 2014; Cakir et al. 2017). Similarly, the changes observed in the present study, including the increased GSH-Px activity in the GH group, may indicate modulation of antioxidant defense mechanisms rather than a direct reduction in oxidative stress.

In this study, serum IL-6 levels differed significantly between groups (*P* < *0.05*), with the C group exhibiting lower values than the GH group at the final sampling. Although IL-6 levels differed between groups, these findings should be interpreted cautiously, as cytokine responses fluctuated over the course of the study. This modulatory effect may be partially mediated by the modulation of inflammatory pathways such as NF-κB. However, these mechanisms were not directly evaluated in the present study, and therefore this interpretation should be considered hypothesis-generating. Bioactive compounds such as organosulfur compounds and flavonoids, have been reported to influence NF-κB activation and pro-inflammatory cytokine transcription (Omonijo et al. 2018). When evaluated together with the hematological findings, these results may suggest a relatively greater inflammatory status in the C group. Although no statistically significant change was observed for TNF-*α* (*P* > *0.05*), a numerical reduction was observed in the treatment groups over time, particularly in those receiving *Hypericum*. Although definitive conclusions regarding cytokine suppression cannot be drawn, the numerical reductions observed in the treatment groups may indicate a moderate anti-inflammatory effect. The anti-inflammatory effect of *H. perforatum* has also been supported by previous studies (İbaokurgil et al. 2022; Heshmati et al. 2018). The lowest proinflammatory response trends were observed in the GH group, which may indicate a potential complementary effect of the two supplements on cytokine responses.Although the effects of black garlic on these cytokines have not been directly investigated in calves, studies in different species report that raw garlic and *Hypericum* can reduce TNF-α and IL-6 levels (Hodge et al. 2002; Kandilarov et al. 2023). These findings are consistent with the partially modulated inflammatory response observed in the treatment groups and may indicate that *Hypericum* contributes to this effect. However, further studies with larger sample sizes are needed to better clarify these responses.

In the present study, C group calves had higher fecal scores throughout the study; this difference was statistically significant on day 45 (*P* < *0.05*). Protocatechuic acid and chlorogenic acid, which are abundant in fermented garlic, have been reported to exhibit synergistic or additive antibacterial effects with antibiotics against multidrug-resistant animal pathogens, particularly Gram-negative bacteria (Chai et al. 2019). Microbiological analyses indicated that neither supplement had a significant effect on *lactic acid bacteria*. However, *bifidobacteria* and *E. coli* counts were significantly lower in the GH group. Black garlic (Botas et al. 2019; Roy et al. 2025) and *H. scabrum *(Engin et al. 2022) have both been reported to exhibit strong antimicrobial activity. *Lactobacillus* species have been reported to be resistant to garlic treatment, while *Bifidobacterium* and other susceptible bacteria are more sensitive (Filocamo et al. 2012; Kubba et al. 2021). These findings suggest a selective antimicrobial effect of the supplements, in which *Lactobacillus spp.* appeared more resistant whereas *Bifidobacterium spp.* were more susceptible. Moreover, the decrease in *bifidobacteria* was more pronounced in the G and GH treated groups, indicating that the antimicrobial activity of garlic is stronger than that of *Hypericum*.

While the reduction in *E. coli* counts reflects a potentially beneficial antimicrobial effect, the decrease in *bifidobacteria* requires careful consideration, given their important role in maintaining gut homeostasis and suppressing pathogenic microorganisms. Therefore, the observed antimicrobial effects cannot be interpreted as entirely beneficial for intestinal health. The reduction in beneficial commensal bacteria suggests that these phytogenic supplements may exert broad-spectrum antimicrobial activity, potentially disrupting microbial balance under certain conditions. *Bifidobacteria* are key contributors to the production of acetate and lactate through carbohydrate fermentation (O’Callaghan and Sinderen, 2016). While acetate is a major short chain fatty acid (SCFA), lactate can serve as a substrate for other commensal bacteria, promoting butyrate production via cross-feeding interactions. These SCFA, particularly butyrate, play a crucial role in intestinal epithelial development, barrier integrity, and immune modulation (Sankarganesh et al. 2025). Importantly, the neonatal period is a critical stage of microbiome development. During this period, microbial succession plays a major role in shaping long-term gut function (Malmuthuge et al. 2015; Dill-McFarland et al. 2019). In addition, the culture dependent techniques applied in this study provide only a partial representation of the intestinal microbiota and may not fully reflect its complexity and diversity. Therefore, interpretations regarding microbial balance, gut ecosystem modulation, and overall intestinal health should be made with caution. Future studies employing sequencing-based approaches such as 16 S rRNA gene sequencing or metagenomics, would enable a more comprehensive evaluation of microbial dynamics and their functional implications.

Various herbal supplements have been shown to improve growth performance and feed efficiency in calves (Zhang et al. 2025; Velázquez-Cruz et al. 2025). In this study, although body weight and measurements were generally higher in the G, H, and GH groups than in the C group, these differences were statistically significant (*P* > *0.05*). While Sorang et al. (2023) reported significant increases in body measurements and weight of pre-weaned calves, Ünlü and Erkek (2013) observed only numerical increases without statistical significance with raw garlic supplementation. At day 60, heart girth measurements were significantly lower in the three treatment groups compared to the C group (*P* < *0.05*). Starter feed consumption did not differ between the groups. Garlic supplementation in ruminant diets has been reported to improve feed palatability, thereby increasing feed intake (El-Naggar and Ibrahim 2018; Martin and Chaudhry 2024). However, no significant differences in starter intake were observed in the present study (*P* > *0.05*), likely because black garlic was administered via milk rather than directly in the feed. FCR and ADG data suggest that both supplements provide modest improvements. However, these benefits were not sustained across all periods and were not reflected in overall body weight or structural growth. The higher FCR and lower ADG values in the C group during specific intervals may be attributed to the combined effects of increased fecal scores. Comparable results have also been reported after raw garlic supplementation (Ghosh et al. 2011; El-Naggar and Ibrahim 2018; Sorang et al. 2023). Although no studies have investigated the effects of *Hypericum* species on calf performance, studies in other farm animals have reported that supplementation wit *H. perforatum* may improve feed efficiency and ADG. (Isık et al. 2024; Ilhan et al. 2024). In the present study, some improvements were observed in certain physiological indicators. However, these changes were not consistently reflected in live weight gain or body measurements. The relatively short experimental duration, the developmental variability of calves, and the limited sample size may have increased inter-individual variation and reduced the statistical power to detect clearer growth responses.

The practical application of these supplements through milk may be feasible under farm conditions, particularly in production systems where calf diarrhea, environmental stress, or early life health challenges are prevalent. However, the present findings remain preliminary and should be validated under larger-scale commercial conditions before definitive practical recommendations can be made. Furthermore, the overall practical and economic value of these supplements depends on balancing the observed improvements in gastrointestinal health indicators against the limited growth responses and reductions in beneficial *bifidobacterial* populations.

## Conclusion

In conclusion, black garlic and *H. scabrum* supplementation influenced several physiological, inflammatory, and gastrointestinal health parameters in pre-weaning calves, including reductions in fecal pathogen counts and in the incidence of diarrhea. However, these effects were accompanied by decreased *bifidobacteria* populations and were not associated with consistent improvements in growth performance. Therefore, while these supplements may show potential as supportive nutritional strategies during the pre-weaning period, the present findings should be considered preliminary and interpreted with caution due to the relatively small sample size and the broad range of evaluated physiological and microbiological parameters. Further studies with larger animal populations, longer experimental periods, and sequencing-based microbiota analyses are needed to provide a more comprehensive understanding of gut microbial dynamics and to validate the microbiological observations reported hereunder commercial calf-rearing conditions.

## Data Availability

The datasets used and/or analyzed during the current study are available from the corresponding author on reasonable request.
